# Endoscopic Management of Large Leakages After Upper Gastrointestinal Surgery

**DOI:** 10.3389/fsurg.2022.885244

**Published:** 2022-05-09

**Authors:** Stanislaus Reimer, Johan F. Lock, Sven Flemming, Alexander Weich, Anna Widder, Lars Plaßmeier, Anna Döring, Ilona Hering, Mohammed K. Hankir, Alexander Meining, Christoph-Thomas Germer, Kaja Groneberg, Florian Seyfried

**Affiliations:** ^1^ Department of Gastroenterology, University Hospital of Würzburg, Würzburg, Germany; ^2^ Department of General, Visceral, Transplant, Vascular and Pediatric Surgery, University Hospital of Würzburg, Würzburg, Germany

**Keywords:** anastomotic leakage, endoluminal, vacuum-assisted closure, negative pressure, endoscopic

## Abstract

**Background:**

Endoscopic vacuum therapy (EVT) is an evidence-based option to treat anastomotic leakages of the upper gastrointestinal (GI) tract, but the technical challenges and clinical outcomes of patients with large defects remain poorly described.

**Methods:**

All patients with leakages of the upper GI tract that were treated with endoscopic negative pressure therapy at our institution from 2012–2021 were analyzed. Patients with large defects (>30 mm) as an indicator of complex treatment were compared to patients with smaller defects (control group).

**Results:**

Ninety-two patients with postoperative anastomotic or staplerline leakages were identified, of whom 20 (21.7%) had large defects. Compared to the control group, these patients required prolonged therapy (42 vs. 14 days, *p* < 0.001) and hospital stay (63 vs. 26 days, *p* < 0.001) and developed significantly more septic complications (40 vs. 17.6%, *p* = 0.027.) which often necessitated additional endoscopic and/or surgical/interventional treatments (45 vs. 17.4%, *p* = 0.007.) Nevertheless, a resolution of leakages was achieved in 80% of patients with large defects, which was similar compared to the control group (*p* = 0.42). Multiple leakages, especially on the opposite side, along with other local unfavorable conditions, such as foreign material mass, limited access to the defect or extensive necrosis occurred significantly more often in cases with large defects (*p* < 0.001).

**Conclusions:**

Overall, our study confirms that EVT for leakages even from large defects of the upper GI tract is feasible in most cases but comes with significant technical challenges.

## Introduction

There is a growing body of evidence showing the remarkable efficacy of endoscopic vacuum therapy (1) to prevent (2–5) or treat (6, 7) anastomotic leakages of the upper GI tract. Overall, EVT has success rates of up to 90% in large meta-analyses (1) and prevents the need for difficult salvage operations that often necessitate demanding secondary reconstructions of the alimentary tract. Thus, EVT has evolved from an experimental procedure to an evidence-based option of choice to treat anastomotic leakages of the upper GI tract in the majority of cases (1). However, the successful application of EVT may come with a significant learning curve (Reimer et al.) associated with various technical challenges and limitations (8), especially in difficult cases. In the present study, we systematically analyzed our prospectively collected database containing detailed information on patients undergoing EVT treatment focusing on patients with large defects (>30 mm) which we considered as a marker for case severity. We then summarize in detail how we overcame the technical challenges we encountered during EVT treatment.

## Materials and Methods

All consecutive patients with leakages of the upper GI tract that were treated with endoscopic negative pressure therapy at our visceral medical center at the University Hospital Wuerzburg, Germany from 2012–2021 were included in this study. Approval was obtained from the local ethics review board (Ethics committee, Würzburg University).

### Study Design and Ethics

For the analysis, all patients with anastomotic or stapler line leakages were included. Patients with large defects (>30 mm) as an indicator of complex treatment were compared to patients with smaller defects (control group). The technical challenges and the evolution of solution being employed were identified, categorized and described in detail.

### Endoscopic Vacuum Therapy

This technique requires a flexible endoscope to place an open-pored polyurethane sponge into the cavity behind the leak (intracavitary) or within the intestinal lumen (intraluminal) (9). The sponge was connected by a nasogastric tube to a negative pressure system. An intracavitary sponge was usually adopted for accessible extraluminal cavities; an intraluminal sponge was generally preferred for defects with diffuse local inflammation or shallow cavities. The sponge was changed regularly every 3–4 days (10). Endoscopic vacuum therapy was terminated when stable granulation tissue was present with no signs of necrosis or leakage.

The vast majority of reported EVT applications at our center was carried out with modified commercially available open-pore polyurethane foam drains that are approved as medical devices for treatment of the esophagus and rectum (EndoSPONGE® and EsoSPONGE®, both B. Braun Melsungen AG, Melsungen, Germany). The modification included removal of the sponge from the original draining tube at the proximal end. The sponge was then carefully cleaned and attached to a 14F gastric tube with 10 perforations on both sides over a length of 6 cm (Vygon, Ecouen, France) with several stitches. A 16F tube was used to drain particularly viscous mucus and a 12F probe was used for angled approaches, smaller cavities, less compliant patients and duodenal lesions. The tip of the tube was snipped off after the sponge was attached to the probe and about 5–7 mm was pulled back into the sponge so that the sponge tip was soft. For localized tissue defects, care was taken to ensure that the suction effect was focused on the defect so that it closed and did not spread to surrounding tissue for avoidance of stricture formation. In our experience, the number and arrangement of the holes on the gastric tube should be limited and restricted to the area carrying the sponge. Therefore, the tube was shortened and additional holes were created on the probe using pliers when necessary (Knipex-Werk C. Gustav Putsch KG, Wuppertal Germany). EndoSPONGE® was used mainly during the first period. In total, <5% of treatments required a sponge longer than 5 cm (V.A.C. Granufoam Dresssing, 3 M, San Antonio, USA or Invia Foam Dressing, Medela, Baar, Switzerland were used).

Foreign body forceps (Rat Tooth Forceps, Endo-Flex GmbH, Voerde, Germany) were applied for endoscopic sponge placement. Standard biopsy forceps and foreign body forceps (Radial Jaw 4, standard capacity, Radial Jaw 4, Jumbo, Boston Scientific, Marlborough, USA and Rat Tooth Forceps, Endo-Flex GmbH, Voerde, Germany) were used for necrosectomy and cleaning the defect margins. In addition, an over-the-scope grasper (OTSG, Xcavator, Ovesco AG, Germany) was occasionally used if extended necrosectomy was necessary. A biliary cytology brush (Cytomax II double lumen, cytology brush, Cook medical, Bloomington, USA) was used to refresh the fistula opening and canal if necessary.

### Statistical Analysis

All statistical analyses were performed using IBM SPSS Statistics 26 (International Business Machines Corporation, Armonk, NY). Descriptive data are reported as means with standard deviations, unless otherwise stated. Comparisons between the analyzed cohorts were performed using chi-square, Fisher’s exact, Mann–Whitney *U*-tests or one-way analysis of variance, in accordance with data scale and distribution. The time-intervals were compared by Kaplan-Meier analysis with log rank test. The level of statistical significance was 0.05 (two-sided).

## Results

Out of 170 patients with EVT for leakages of the upper GI tract including several entities, 92 patients with a postoperative anastomotic or staplerline leakage were identified. Of those, 20 patients (anastomotic leak *n* = 16 and staplerline leak *n* = 4) with large defects (>30 mm) were detected and compared to patients with smaller defects (*n* = 72). Baseline characteristics are summarized in **Table 1**.

**Table 1:** Patient and leakage characteristics.

Characteristic	Patients, No. (%)		
	Large defects (*n* = 20)	Control (*n* = 72)	*p* value
Sex ratio, No. (M:F)	13:7	59:33	.94
Age, mean (SD), y	60.7 (8.8)	58.8 (14.1)	.57
BMI, mean (SD), kg/m^2^	27.8 (5.5)	28.3 (9.5)	.80
Charlson comorbidity index, mean (SD)	3.8 (2.3)	4.2 (2.5)	.52
ASA classification ≥III	14 (70.0)	62 (67.4)	.82
Neoadjuvant therapy	7 (35.0)	44 (47.8)	.19
Oncological surgery	9 (56.3)	49 (63.6)	.58
UGI surgery	7 (43.8)	28 (36.4)	
Type of leakage			
Esophago-gastrostomy	8 (40.0)	33 (35.9)	.34
Esophago-jejunostomy	2 (10.0)	28 (30.4)	
Gastro-jejunostomy	5 (25.0)	17 (18.5)	
Other	5 (25.0)	14 (15.2)	
Interval from surgery to diagnosis of leakage, mean (95%CI), d	8.8 (4–22)	11.5 (2–31)	.27
Initial leakage diameter, mean (95%CI), mm	24.5 (17.7–31.2)	7.4 (6.2–8.6)	<.001

*Values are n (%) unless otherwise indicated.*

*UGI, upper gastrointestinal tract; SD, standard deviation; 95%CI, 95% confidence interval.*

**Figure 1:**
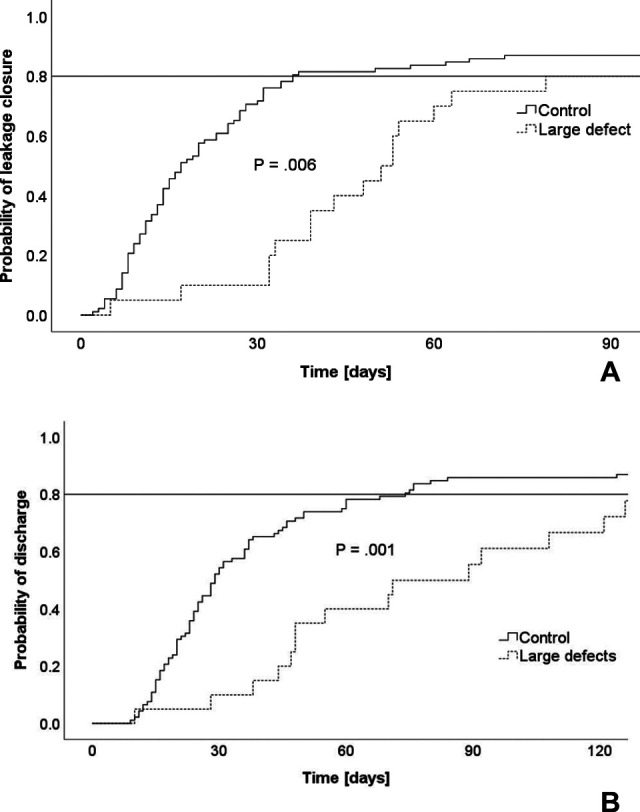
Impact of large defects on recovery. (**A**) Duration of leakage therapy. (**B**) Length-of-hospitalization.

Baseline characteristics including age, gender, comorbidities, entity and side of the leakage did not differ between both groups. Patients with a large defect size of the leakage (with an estimated size of over 30 mm or half of the anastomotic circumference, respectively) had prolonged treatment duration (**Figure 1**). Treatment outcomes are summarized in **Table 2**. Patients with larger defect sizes required prolonged therapy and, consequently, experienced extended hospital stay. Compared to the control group, they developed significantly more septic complications and more often required additional endoscopic and/or surgical/interventional treatments. Nevertheless, leakages resolved in 80% of patients with large defects compared to 90% of patients in the control group, which was not significantly different.

**Table 2:** Endoscopic leakage therapy and outcome.

Characteristic	Patients, No. (%)		
	Large defects (*n* = 20)	Control (*n* = 72)	*p* value
Duration of leakage therapy, median (quartiles), d	42 (32–54)	14 (8–25)	<.001
Sponge changes, median (quartiles)	12 (10–16)	4 (2–6)	<.001
Challenging endoscopic situations			
Leakage with >1 defect	10 (50)	5 (5.4)	<.001
Foreign material within leakage	10 (50)	2 (2.2)	<.001
Limited endoscopic access to leakage	10 (50)	3 (3.3)	<.001
Extensive necrosis	12 (60)	6 (6.5)	<.001
Additional Procedures during EVT			
Any reoperation	10 (50)	24 (26.1)	.035
Percutaneous abscess drainage	8 (40)	15 (16.3)	.017
Complications during EVT			
Recurrent sepsis	8 (40)	16 (17.6)	.027
Stenosis/ stricture	4 (20)	12 (13)	.48
Efficacy of EVT			
Improvement of leakage	18 (90)	82 (89.1)	.91
Resolution of leakage	16 (80)	80 (87.0)	.42
Resolution without additional procedures during or after EVT	9 (45)	63 (68.5)	.047
Failure-to-cure^a^	3 (15)	10 (10.9)	.61
Additional Procedures after EVT	9 (45)	16 (17.4)	.007
Endoscopic clip	4 (20)	12 (13.0)	.42
SEMS	5 (25)	4 (4.4)	.002
In-hospital mortality	2 (10)	6 (6.5)	.58
Length-of-stay, median (quartiles), d	63 (45–104)	26 (18–45)	<.001
Oral nutrition on discharge	14 (70)	70 (76.1)	.57

*Values are n (%) unless otherwise indicated.*

*EVT, endoscopic vacuum therapy; 95%CI, 95% confidence interval; SEMS, self-expanding metal stent.*

^*a*^ *Conversion to surgical therapy due to deteriorating leakage during EVT or death.*

### Challenging Endoscopic Situations and Proposed Solutions

**Table 3** summarizes the main technical challenges associated with EVT and the proposed solutions. Multiple leakages, especially on the opposite side, along with other local unfavorable conditions, such as foreign material mass, limited access to the defect or extensive necrosis was found significantly more often in cases with large defects (85% vs. 14.1%, *p* < 0.001, **Table 2**).

**Table 3:** Challenging endoscopic situations and proposed solutions.

Technical challenges	Problem	Proposed solution
Leaks with more than one (deep) defect	Intraluminal EVT may be ineffective to sufficiently drain deep defects	Intracavital sponge placement by applying two or more sponge systems
Foreign material mass	Foreign material may preclude sufficient suction and/or collapse of the defect	Extracting foreign material whenever possible
Limited access to the leak/defect (small caliber, tissue bridges, deep cannels)	Inefficient suction/drainage of the defect	Optimizing access to the defect (e.g., by tissue dissection, pneumatic dilatation (11), creation of alterative routes (e.g., stoma formation, (12))
Extensive necrosis at leak/defect site	EVT induced tissue granulation needs vital tissue	Early and extensive necrosectomy

**Figure 2:**
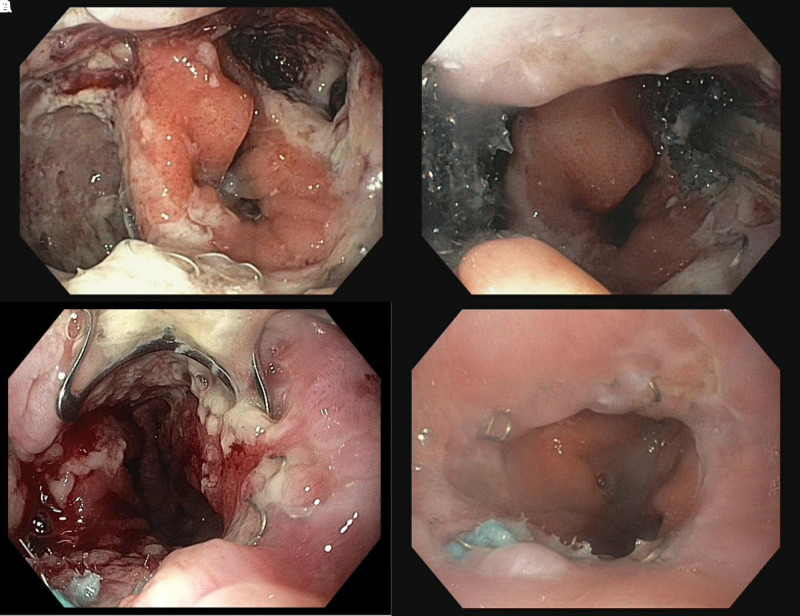
(**A–D**) Usage of two independent sponge systems in a case with two large defects of the oseophagojejunostomy (Merendino Procedure) and results. Initial endoscopy showed an evident anastomotic leakage between 02:00 and 05:00 and between 08:00 and 11:00. After extensive necrosectomy on both leakages (**A**), two separate sponge systems were inserted into both insufficiency cavities (**B**). In order to accelerate further healing and to minimize the risk of fistula formation, the healed leakage cavities were gathered using mini OTSC, 8 mm (**C**) and an intraluminal sponge was inserted. Re-endoscopy of the anastomotic region after 14 days (**D**). OTSC is completely grown into the wall. Anastomosis is largely free of irritation.

**Figure 3:**
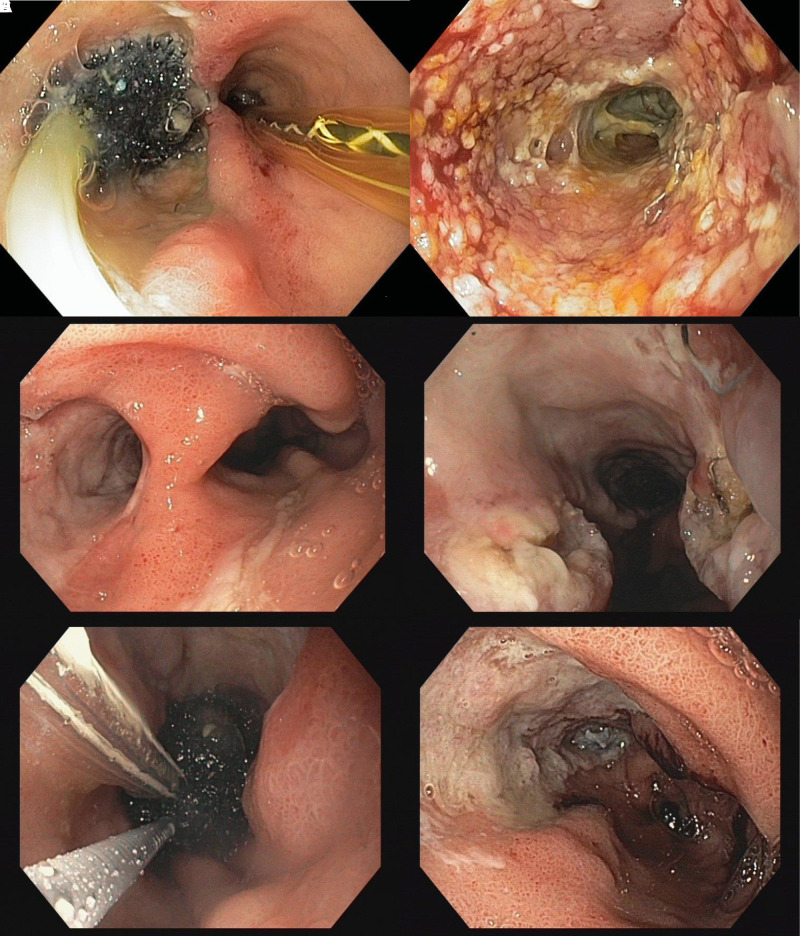
(**A–F**) Creation of an optimal access route to the leakage defect in a patient after sleeve gastrectomy. Endoscopic pictures of a female patient with a chronic large defect of the proximal stapler line after sleeve gastrectomy transferred to our institution for complication management. Initial endoscopy found a 40 × 15 mm long EVT sponge in an approx. 12 cm long and 15mm wide paragastral defect. A gastric tube was placed intraluminary of the sleeve stomach (**A**). In the area of the defect ground, no suction marks but necrosis and fibrin deposits were detected (**B**). Necrosis and fibrin were removed using forceps and a brush. In a further step, in order to enable wide endoscopic access to the defect ground the canal was opened towards the gastric tube using a clutch cutter (Fjuifilm). (**C,D**) After further EVT (**E**), a gastric tube with a continuous lumen of approx. 4 cm is found. The approximately 12 cm long former defect canal is completely epithelialized in the proximal half and almost completely epithelialized in the distal area (**F**).

**Figure 4:**
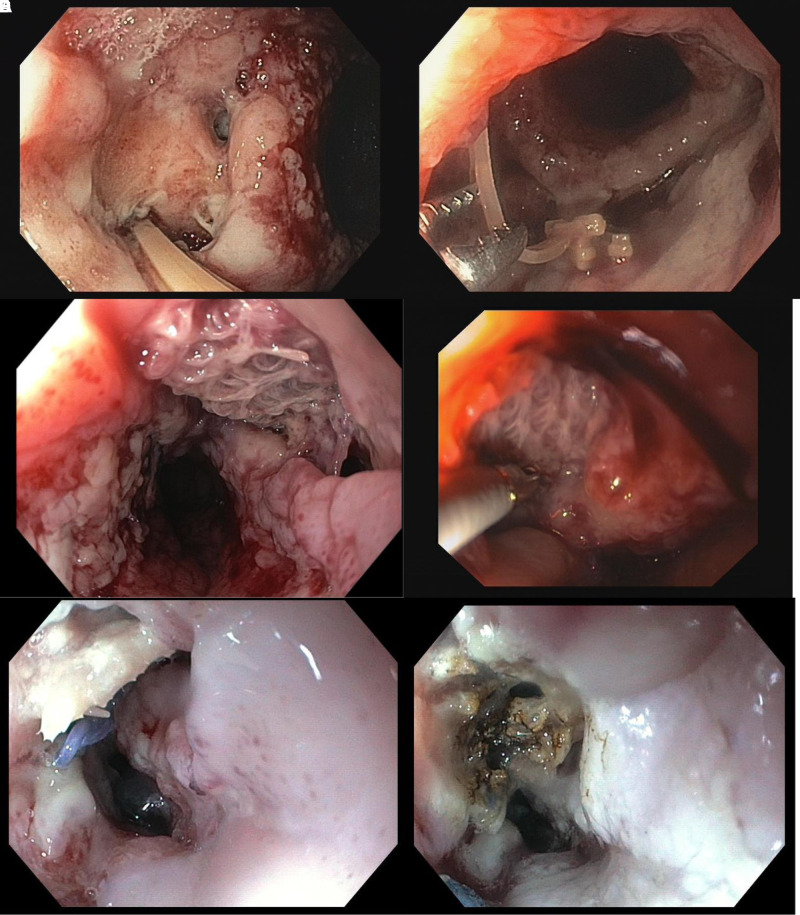
(**A–F)** exemplary presentation of foreign material at the anastomotic leak site. Leak of the oesophagogastrostomy after minimally invasive Ivor Lewis surgery. Detectable vessel clip on the azygos vein (**A**). Removal of vessel clips using forceps (**B**). Leakage of the esophagus after revisional hiatal surgery with mesh augmented hiatoplasty (**C**, **E**). Partial repositioning (**D**) and status after thermal destruction of the intraluminal mesh portion by argon plasma coagulation (**F**).

**Figure 5:**
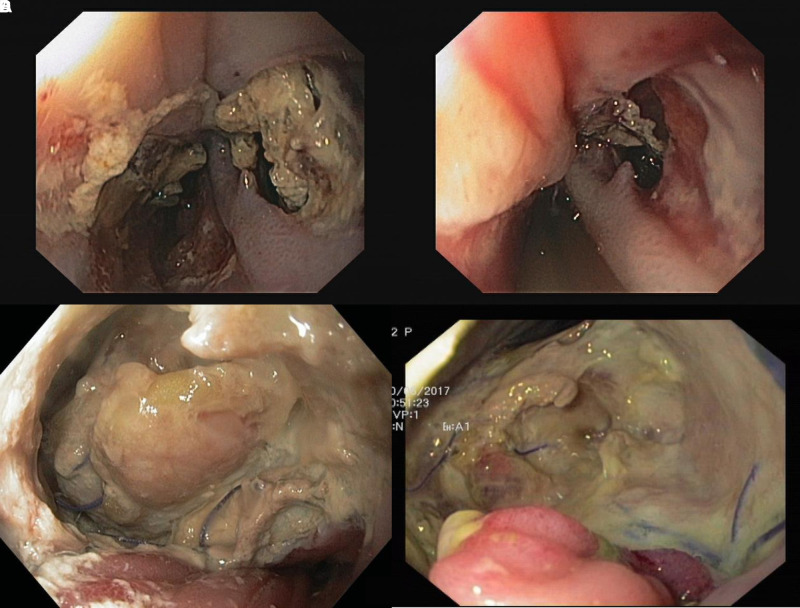
(**A–D**) Endoluminal view of an esophageal defect with tissue necrosis. Initial necrosis (**A**, **C**) and condition after necrosectomy (**B**, **D**). It can be seen after necrosectomy that the transmural portion of necrosis is smaller than initially assumed.

Within this group the majority of patients even showed multiple endoscopic difficulties (75% vs. 3.3%, *p* < 0.001). We found an association of the number of challenging endoscopic situations and the median duration of EVT (none: 12 days, 1 difficulty: 27 days, >2 difficulties: 42 days, *p* < 0.001).For these problems, several solutions were identified and successfully applied in our patients. **Figures 2–5** demonstrate the endoscopic management of these challenging endoscopic situations.

## Discussion

Surgical options available for the management of complex leakages of the upper GI tract are limited and usually contain a high risk of morbidity and mortality (10). Our results confirm previous findings on the effectiveness of EVT for treating anastomotic and suture-line leakages of the upper GI tract (1).

In this study, we focused on the technical challenges and clinical outcomes of patients with large defects. We found that endoscopic management of large leakages after upper GI surgery is feasible but contains technical challenges which need to be mastered to achieve good results.

Interestingly, baseline patient characteristics of the group with larger defects were not different to the control group with smaller leakages. While consistent risk factors for the development of an anastomotic or stapler line have been reported (13), our data does not provide further insights on which patients may develop larger defects leading to a more challenging course. Larger defects may have occurred due to insufficient perfusion of the anastomotic region, even though mucosal signs of ischemia during endoscopic treatment were not detected. Combined hyperspectral imaging (HIS) or florescence Imaging (FI) with indocyanine green (ICG) were not routinely performed but could provide further insights in the future.

It could also be possible that a delayed start or initially insufficient treatment of the leakage may have contributed to a larger defect size (14) as some of the patients treated at our tertiary hospital underwent surgery elsewhere and were transferred for leakage management during the later course.

We previously showed that experience with EVT in conjunction with adjustments in institutional factors, patient management and technical details positively impact on its overall efficacy (Reimer et al). Given the remarkable success rates of EVT, it seems reasonable to implement this promising technique for more complex cases.

The successful treatment of large defects contains some technical challenges which frequently occur during treatment. Of note, it is not unusual that the initial defect size increases during early treatment.

If a leakage with more than one deep defect with spatial distance to each other occurs, we recommend the usage of more than one sponge system so that an intracavital placement is possible to sufficiently drain all defects. Foreign material may preclude sufficient suction and the collapse of the defect and should therefore be removed. Extensive necrosis at the anastomotic leakage site should also be removed as early as possible since EVT induced tissue granulation needs healthy tissue (15). If access to endoscopic treatment is limited, several options can be considered including tissue dissection, dilatation or creation of alternative routes (11, 12). When these principles are applied, there are only very few conditions where an EVT does not provide good outcomes.

Whenever a difficult leakage is treated by EVT, it is extremely important to evaluate carefully and constantly both, the local leak situation but, more importantly, the patient’s systemic condition. An interdisciplinary board of experienced gastroenterologists and visceral surgeons should consider alternative endoscopic or surgical treatment options whenever necessary (8, 16).

Our results show that patients with larger defect sizes needed prolonged therapy. Compared to the control group, they developed significantly more septic complications and required more often additional endoscopic and/or surgical/interventional treatment. However, also in this cohort a resolution of the leakage was achieved in 80%, with an improvement in 90% of patients, respectively. Thus, neither the success nor the mortality rates were different compared to the control group.

This is to our knowledge is the first study comparing patients with large defects to patients with small anastomotic leakages. A limitation may be the small number of patients with large leakages. Nevertheless, this is one of the largest prospectively collected databases focusing on EVT treatment for more than 10 years. Due to the small number, we may have missed the opportunity to detect some other potential differences with the control group because of statistical power. Additionally, it is difficult to systematically categorize all of the technical challenges which may occur during EVT treatment either alone or even in combination.

In summary, our study confirms that EVT for leakages even with large defects in the upper GI tract is successful in the vast majority of cases but contains some technical challenges which need to be addressed.

## Data Availability

The original contributions presented in the study are included in the article/supplementary material, further inquiries can be directed to the corresponding author/s.
